# Novel Widespread Marine Oomycetes Parasitising Diatoms, Including the Toxic Genus *Pseudo-nitzschia*: Genetic, Morphological, and Ecological Characterisation

**DOI:** 10.3389/fmicb.2018.02918

**Published:** 2018-12-03

**Authors:** Andrea Garvetto, Elisabeth Nézan, Yacine Badis, Gwenael Bilien, Paola Arce, Eileen Bresnan, Claire M. M. Gachon, Raffaele Siano

**Affiliations:** ^1^The Scottish Association for Marine Science, Scottish Marine Institute, Oban, United Kingdom; ^2^IFREMER, ODE/UL/LER BO, Station de Biologie Marine de Concarneau, Concarneau, France; ^3^Marine Scotland Science, Marine Laboratory, Aberdeen, United Kingdom; ^4^IFREMER – Centre de Brest, DYNECO PELAGOS, Plouzané, France

**Keywords:** *Pseudo-nitzschia*, diatoms, marine oomycetes, plankton parasites, single-cell analysis, metabarcoding

## Abstract

Parasites are key drivers of phytoplankton bloom dynamics and related aquatic ecosystem processes. Yet, the dearth of morphological and molecular information hinders the assessment of their diversity and ecological role. Using single-cell techniques, we characterise morphologically and molecularly, intracellular parasitoids infecting four potentially toxin-producing *Pseudo-nitzschia* and one *Melosira* species on the North Atlantic coast. These sequences define two, morphologically indistinguishable clades within the phylum Oomycota, related to the genera of algal parasites *Anisolpidium* and *Olpidiopsis* and the diatom parasitoid species *Miracula helgolandica*. Our morphological data are insufficient to attribute either clade to the still unsequenced genus *Ectrogella*; hence it is proposed to name the clades OOM_1 and OOM_2. A screening of global databases of the barcode regions V4 and V9 of the 18S rDNA demonstrate the presence of these parasitoids beyond the North Atlantic coastal region. During a biweekly metabarcoding survey (Concarneau Bay, France), reads associated with one sequenced parasitoid coincided with the decline of *Cerataulina pelagica* bloom, whilst the other parasitoids co-occurred at low abundance with *Pseudo-nitzschia*. Our data highlight a complex and unexplored diversity of the oomycete parasitoids of diatoms and calls for the investigation of their phenology, evolution, and potential contribution in controlling their host spatial-temporal dynamics.

## Introduction

Parasitism is regarded as one of the most widespread ecological strategies among heterotrophs (Lafferty et al., 2006, 2008). Early marine and freshwater metagenomic surveys revealed a broad diversity of planktonic parasites in aquatic systems, with up to 20% or 30% of OTUs clustering within parasitic groups of marine dinoflagellate or freshwater zoosporic fungi, suggesting that planktonic protists are no exception to this rule (Guillou et al., 2008; Lepère et al., 2008). More recently, OTUs related to known parasites contributed to over 50% in richness and abundance of the heterotrophic pico-nanoplankton (0.8–5 μm) sequenced during the *Tara Oceans* expedition, and parasitic interactions dominated plankton networks (de Vargas et al., 2015; Lima-Mendez et al., 2015). While the presence and distribution of presumed parasites as well as the correlation with putative hosts can be inferred by taxonomic annotation of metagenomics/metabarcoding data (e.g., Taylor and Cunliffe, 2016; Mahé et al., 2017), the characterisation of novel organisms and the host–parasite relationship need specific, *in vivo* observations to validate *in silico* inferences (Figueroa et al., 2010). All main phytoplankton groups are targeted by viruses, algicidal bacteria and protistan parasites (Gachon et al., 2010). These organisms are often referred as parasitoids since they usually kill their unicellular host, and directly influence the dynamics of their host population (Kühn et al., 2004). They are most often reported when remarkably high prevalence of infection is linked to population crashes. For example, Sparrow (1969) recorded 99% prevalence of infection by the oomycete *Ectrogella perforans* in the marine epiphytic diatom *Licmophora* sp. in the north-west coast of the United States. In lentic ecosystems, a similarly high prevalence of chytrids (>90%) is often reported during blooms of the diatom *Asterionella formosa* and the cyanobacteria *Anabaena* sp. (e.g., Van Donk and Ringelberg, 1983; Rasconi et al., 2012). Aside from these extremes, however, bloom demise and top-down control of microphytoplankton by eukaryotic parasitoids is achieved with much lower infection prevalence, for diatoms (Tillmann et al., 1999; Peacock et al., 2014) as well as dinoflagellates (Montagnes et al., 2008; Siano et al., 2011; Velo-Suárez et al., 2013), in both marine and freshwater habitats (Holfeld, 1998; Alster and Zohary, 2007). Some parasitoid dinoflagellates are even thought to govern the temporal succession of blooming phytoplankton (Chambouvet et al., 2008). Diatoms are infected by many small flagellates as diverse as dinoflagellates, rhizarian, aphelida, and stramenopiles such as hyphochytrids and labyrinthulomycetes (Scholz et al., 2016a and references therein). Some of the best known parasitoids of diatoms belong to the Chytridiomycota (Fungi) and Oomycota (Heterokontophyta), both in freshwater and in the sea (Scholz et al., 2016b; Frenken et al., 2017). Among oomycetes, four taxa are classically known to infect diatoms, all of them endobiotic (reviewed in Dick, 2001). *Lagenisma coscinodisci*, is the only species that produces hyphae and was characterised molecularly as an early diverging member of the crown oomycetes (*sensu* Beakes et al., 2012), a group that encompasses well-studied land- and freshwater-dwelling saprobes and parasites of plants and animals (Thines et al., 2015). The genus *Ectrogella* Zopf emend. Scherffel regroups several species of intracellular, unbranched, holocarpic pathogens of freshwater and marine, centric and pennate diatoms; *Ectrogella* shares many morphological features with *Olpidiopsis*, which led to the transfer of the parasitoid of *Pleurosigma attenuatum* and *Nitzschia* sp. from *Olpidiopsis gillii* to *Ectrogella bacillariacearum* (Dick, 2001). Finally, *Lagenidium enecans* and *Aphanomycopsis bacillariacearum*, both forming resting spores reminiscent of those found in other Lagenidiales, are only known through sporadic microscopy observations of field-collected material. *Lagenisma coscinodisci* and *Ectrogella perforans* are the only species whose ultrastructure and development cycle are relatively detailed (Schnepf and Deichgräber, 1978; Raghu Kumar, 1980a,b). Amongst diatoms, the toxigenic genus *Pseudo-nitzschia* is considered ecologically and economically important because it affects human health, coastal aquaculture industries and can negatively impact wildlife such as sea birds and mammals (Lelong et al., 2012; Trainer et al., 2012). Some species within this genus are responsible for Amnesic Shellfish Poisoning (ASP), due to the production of domoic acid and its isomers, neurotransmitter-like amino acids which enter the food web through filter feeders, and can affect marine birds, marine mammals and humans that feed upon the contaminated seafood (Trainer et al., 2012). Twenty-six species within the genus *Pseudo-nitzschia* are potentially responsible for ASP according to the Taxonomic Reference List of Harmful Micro Algae from the Intergovernmental Oceanographic Commission of UNESCO (Moestrup et al., 2009). Domoic acid-producing *Pseudo-nitzschia* have been, with few exceptions, linked to a cosmopolite geographical distribution on a species level (Hasle, 2002), although different isolated molecular clades can exist (Casteleyn et al., 2010). Toxin-producing blooms are most often reported from coastal areas, especially in upwelling zones (Lelong et al., 2012). Two parasitoids have been observed on *Pseudo-nitzschia*, an unassigned chytrid and an oomycete tentatively affiliated to the genus *Ectrogella* according to its morphology (Hanic et al., 2009). The oomycete from Prince Edward Island (Canada) infected *Pseudo-nitzschia pungens* during the autumn bloom in the years 1992–1995 and 2008. The prevalence of infection ranged from 0.6 to 15.9% of the host population depending on the sampling site and period. Morphologically similar parasitoids of *Pseudo-nitzschia* were also reported in the United States and Scotland (Gachon et al., 2010; Lelong et al., 2012; Trainer et al., 2012), suggesting a wide dispersal of these oomycetes across the Northern hemisphere. Recently, two oomycete parasitoids infecting *Pseudo-nitzschia pungens* (Prince Edwards, Canada and Helgoland, Germany) and *Rhizosolenia imbricata* (Helgoland, Germany) were described as two new species, *Miracula helgolandica* and *Olpidiopsis drebesii* on the basis of short sequences (ca. 500 bp) of the 18S rDNA (Buaya et al., 2017). Here, we report the morphological and molecular characterisation of seven oomycete parasitoids of *Pseudo-nitzschia* spp. and *Melosira*, and use environmental DNA sequences to assess their widespread occurrence and their dynamics in the plankton community.

## Materials and Methods

### Sampling and Microscopy Observation

Infected diatoms were isolated from near surface seawater samples collected with a 20 μm mesh size net tow, or from tidal pools. Water samples were immediately screened and/or stored at 9°C in 50 mL vented cell culture flasks and analysed within the following 10 days with a Zeiss Axiovert 200 inverted microscope or preserved with acidic Lugol’s Iodine solution (0.1% final concentration) and stored at 4°C until examination. Single infected cells or clonal chains were identified (Tomas et al., 1997), isolated by mouth pipette and transferred in single drops on a glass coverslip for microscopy (Zeiss AxioObserver inverted microscope, Oberkochen, Germany or Olympus IX70 inverted light microscope equipped with a digital camera DP72, Olympus, Tokyo, Japan). Specimens were photographed, rinsed twice with double distilled water (Millipore filtered water) or filtered-autoclaved seawater, transferred in sterile microcentrifuge tubes and stored at -20°C awaiting whole genome amplification or nested PCR amplification followed by cloning. Some empty sporangia were isolated as above and stained in the dark for 15–30 min with 50 μg/mL Calcofluor-White, before being examined using both bright field and epifluorescence microscopy. The complete list of samples and GPS coordinates of sampling points is given in Table 1, whilst details on PCR primers used in this study are specified in Supplementary Table S1.

**Table 1:** Details of the single-cell samples and molecular markers retrieved this study.

Sample ID	Date	Host	Pictures	Location	Amplification	Primer	Primer	Primer	Molecular	GenBank
				(lat.–long.)	method	set 1	set 2	set 3	marker	accession number
10-044	17/03/2010	*P. australis*	Figure 1B and Supplementary Figure S5A	N 45°47’59”	Nested PCR	18SFW	18SFW	18SF7	18S	MF960901
				W 01°12’19”		18SRV	18R7	18SRV		
10-045	17/03/2010	*P. australis*	Supplementary Figure S5B	N 45°47’59”	Nested PCR	18SFW	18SFW	18SF7	18S	MF960902
				W 01°12’19”		18SRV	18R7	18SRV		
12-150	17/07/2012	*P.* cf. *plurisecta*	Supplementary Figure S5C	N 48°27’0”	Nested PCR	18SFW	18SFW	SR9FW	18S	MF960904
				W 05°06’54”		18SRV	1250R	18SRV		
13-374	09/09/2013	*P. fraudulenta*	Supplementary Figure S5D	N 48°18’34”	Nested PCR	18SFW	18SFW	1050F	18S	MF960905
				W 04°26’55”		18SRV	SR9PR	18SRV		
14-236	08/09/2014	*P. pungens*	Supplementary Figure S5E	N 48°18’34”	Nested PCR	18SFW	18SFW	SR9FW	18S	MF960906
				W 04°26’55”		D3B	SR9PR	28KARREV		
Ect6para	07/09/2016	*P. australis*	None	N 56°27’12”	MDA/PCR	F139	None	None	18S	MF960903
				W 5°26’10”		R1233				
					MDA/HiSeq 3000	None	None	None	cox2	MG787100
Melo1para	06/12/2016	*M.* cf. *nummuloides*	Figures 2A,C–F and Supplementary Figure S5F	N 56°26’56”	MDA/PCR	F139	None	None	18S	MF960907
				W 5°26’9”		R1233				
					MDA/HiSeq 3000	None	None	None	cox1	MF960908
									cox2	MF960909

### DNA Amplification Methods

Whole genome amplification through Multiple Displacement Amplification (MDA; Lasken, 2007) of samples Ect6Para and Melo1Para (Table 1) was carried out with the Qiagen REPLI-g^®^ Single Cell Kit, as per the manufacturer’s instruction. Typically, 32 μg DNA/40 μl were obtained per each sample and diluted 1:100 (V:V) for downstream PCR amplification with the primer set F139-R1233 (Supplementary Table S1), targeting ca. 900 bp within the 18S rDNA gene of Oomycetes. PCRs took place in a total reaction volume of 50 μL containing 3 μL of genomic DNA, 1 μl (0.2 ng/μl) of each primer, 25 μl of master mix solution (Taq PCR Mastermix, Qiagen) and 20 μl of sterile Millipore filtered water. PCR conditions were as follows: 94°C for 4 min, followed by 35 cycles of 94°C for 1 min, 54°C for 1 min 30 s and 72°C for 2 min. An elongation step of 5 min at 72°C completed the cycle. PCR products were purified on gel with the GeneJET Gel Extraction (Thermo Scientific) and DNA clean-up Micro Kit (Thermo Scientific), followed by Sanger sequencing (GATC Biotech, Konstanz, Germany). Single cell samples 10-044, 10-045, 12-150, 13-374 and 14-236, were used as template for nested PCRs. Almost the full length of the 18S rDNA was amplified with the primers 18S-FW and 18S-RV (Grzebyk et al., 1998) or D3B (Nunn et al., 1996). For sample 14-236 PCR amplification was carried out with the same primers, but the retrieved sequence was shorter (1,180 bp) and contained 73 unassigned positions. PCR products obtained were used as templates for a second step of amplification with different primers depending on the specimen (Table 1 and Supplementary Table S1). PCRs were performed in 25 μL reactions containing 12.5 μL of 1X PCR master mix (Promega), 2.5 μL (10 μM) of each primer and 6.5 μL of water. PCR conditions were 94°C for 2 min, followed by 45 cycles of 94°C for 30 s, 52–56°C (depending on the primer set used) for 1 min, 72°C for 4 min and a final extension step of 72°C for 5 min. As a quality check, PCR products were run at 120 V for 50 min on agarose (1%) TAE buffer (1x) and stained with ethidium bromide. PCR products were purified with a Wizard^®^ SV Gel and PCR Clean-Up System Kit (Promega) according to the manufacturer’s recommendations. Products were sequenced using the ABI PRISM BigDye Terminator Cycle Sequencing kit (Applied Biosystems, Carlsbad, CA, United States). Sequencing products were purified by exclusion chromatography using the Dye Terminator Removal kit (Abgene Ltd., Epsom, United Kingdom) and the sequences were determined using an automated 3130 genetic analyser (Applied Biosystems). For sample 14-236 the composition of the PCR reaction differed, being 7.8 μL of PCR Grade water, 0.6 μL of each primer and 10 μL of Kod Ho Start Master Mix DNA polymerase (Merck Millipore). Downstream manipulations follow the above description. Furthermore aliquots of the MDA amplification products of samples Melo1Para and Ect6para were used to construct a genome library NEBNext Ultra DNA Library Prep Kit for Illumina, followed by sequencing on Hiseq 3000 150 bp paired end. Paired-end reads were merged and quality trimmed in CLC-Genomics Workbench 8 (CLCbio, Qiagen, Hilden, Germany). Reads matching the *cox1* and *cox2* genes were retrieved by tblastn and assembled *de novo* in Geneious 6.1.8 (Kearse et al., 2012). The contigs were curated manually to remove contaminants and any chimaera retrieving *cox1* for Melo1para and *cox2* for both Melo1para and Ect6para.

### Sequence Analysis and Phylogenetic Reconstruction

Sanger-sequences of the total or partial 18S rDNA of the specimens studied were assembled with Geneious 6.1.8 (Kearse et al., 2012). Novel sequences were added to the alignment of Gachon et al. (2017), together with other oomycete sequences from GenBank, realigned with MAFFT (Katoh et al., 2002), manually curated and trimmed, for a total of 81 sequences. Poorly aligned positions were removed with Gblocks 0.91b (Castresana, 2000; parameters in Supplementary Table S2), which resulted in a 37% reduction in the length of the aligned sequences (from 1,896 bp to 1,206 bp). The dataset was then used to compute NJ (1,000 bootstraps) tree using the Tamura-Nei genetic distance model. A tree following the MP (100 bootstraps) criterion was computed using MEGA 7.0.14 (Tamura et al., 2007) default parameters. IQ-TREE 1.5.5 (Nguyen et al., 2015) was used to calculate a ML (1,000 bootstraps) phylogeny after assessment of the best fitting model of molecular evolution via ModelFinder (Kalyaanamoorthy et al., 2017), resulting in TIM2+R4. As a control the manually curated alignment before automated Gblock trimming (1,344 positions) was used to estimate a phylogeny with ML and NJ methods as described above (Supplementary Figure S1 and Supplementary Data Sheet S1). To further support the 18S based phylogeny, the amino acid sequences of the mitochondrial markers *cox1* (260 aa) and *cox2* (191 aa) for the parasitoid of *Melosira* cf. *nummuloides* were concatenated and aligned with a dataset of 25 oomycetes *cox1* and *cox2*. Four diatoms were selected as an outgroup. For the parasitoid Ect6para isolated in *P. australis* only the *cox2* (156 aa) was retrieved and added to the respective alignment. ML reconstruction was performed in IQ-TREE and the best fitting model assessed through ModelFinder for the two protein alignments separately. The two best fitting models, i.e., mtZOA+G4 for *cox1* and mtZOA+F+G4 for *cox2* (Rota-Stabelli et al., 2009), were combined in a partition model (Chernomor et al., 2016) allowing each protein to have its own evolution rate (IQ-TREE option -spp). NJ reconstruction under Tamura Nei distance model was also calculated, as described above. Results are shown in the phylogenetic tree in Figure 4.

### Assessment of OOM_1 and OOM_2 Global Distribution and Diversity

The 18S rDNA sequences from samples 12-150, 13-374, 10-044, and 10-045 were used as a query to investigate the metabarcode datasets from BioMarKs (Logares et al., 2014), Ocean Sampling Day (Kopf et al., 2015) and TARA Ocean^1^ (de Vargas et al., 2015). *Pseudo-nitzschia australis* parasitoids 10-044 and 10-045 were merged into a single query, because their 18S rDNA V4 – V9 hypervariable regions are identical. 467 runs of the Ocean Sampling Day project (PRJEB8682, 162 biosamples) were screened using EDirect and SRA Toolkit utilities combined with in-house scripts referred to as MOULINETTE (Badis et al., *subm.*). MOULINETTE extracts from metabarcoding projects deposited in the Sequence Read Archive all reads (if any), that match the given query sequence, and their GPS coordinates. For the TARA Ocean and BioMarKs datasets, a blastn of, respectively, the V9 and the V4 hypervariable region was conducted (Altschul et al., 1990). Stations were considered positive and GPS points retained and mapped if they contained 10 or more merged paired reads with over 99% identity to the V4 – V9 hypervariable region of the query sequence of our diatom parasitoids. Maps were generated using the open source software GPS Visualizer^2^ (Adam Schneider). The global distribution and diversity of the OOM_1 and OOM_2 clades were also investigated. Reads related to all the sequences of our parasitoids were further clustered into OTUs and their presence in metabarcode databases screened. For this purpose, SRA was screened using MOULINETTE for 1127 publicly available marine metabarcoding datasets using the hypervariable region V4 of the 18S rDNA. MOULINETTE used a relaxed approach, i.e., reads were retained when at least 97% identical over 80% of their length to the query sequence. Paired reads were filtered (expected error over 1.0) and clustered using USEARCH (v9.1.13, Edgar, 2010). All OTUs were then aligned to reference oomycete sequences, including the parasitoids described here, using MUSCLE. A ML tree was inferred using MEGA 7.0.14 (100 Bootstrap; T3G parameter; 70% site coverage cutoff) and the OTUs clustering with our reference diatom parasitoid sequences were selected. Each read was assigned a best-matching OTU using blastn, and the distribution of each OTU was plotted in GPS Visualizer using GPS coordinates of each read with over 97% identity. Furthermore the retrieved OTUs were aligned to our reference backbone tree of oomycetes with the same parameters used above. The resulting alignment was used to compute a ML tree in MEGA 7.0.14 with bootstrap test of phylogeny set to 1000, using the best fitting model Tamura 3 parameters and gamma distribution (four categories) and 80% coverage cut-off. A second screening of SRA deposited metabarcoding databases was done to exclude the presence of the studied parasitoids in freshwater environments. Metabarcode datasets were selected using the keywords “Freshwater,” “Lake,” “Wastewater,” and “Aquatic” (see Badis et al., *subm.* for details) resulting in the screening of 19,000 datasets (data not shown).

### Assessment of Local Ecological Dynamics

Water was sampled with Niskin bottles and fractionated via sequential filtration upon 20 μm, 3 μm, and 0.2 μm mesh-size polycarbonate filters. For the two larger size fractions, water was driven through the filter by a peristaltic pump until filter clogging, resulting in variable amounts of filtered sea water (1.6–11.2 L). For the smallest filter, 500–1,000 mL of residual filtrate from the previous steps was used. Slightly disagreeing from the definition of size fractions proposed by Sieburth and Smetacek (1978), plankton size categories are here named: micro- (>20 μm), nano- (20-3 μm) and picoplankton (3–0.2 μm). After filtration, filters were immediately frozen in liquid nitrogen and then stored at -80°C awaiting analysis. The 18S rDNA V4 hypervariable region was chosen as genetic barcode, since this marker has been acknowledged to give good taxonomical resolution to distinguish unicellular eukaryotes (Stoeck et al., 2010; Behnke et al., 2011; Dunthorn et al., 2012; Pawlowski et al., 2012). Genomic DNA was extracted following the DNA extraction kit Nucleospin Plant II (Macherey-Nagel, Hoerdt, France). As different water volumes were sampled, final DNA concentration was normalised to 5–10 ng/μL of all extracts. In parallel, to check and validate the extraction procedure, some blank extractions (Millipore filtered water) were carried out. DNA quality (proteins/DNA absorbance: A260/A280) and concentration of purified products was, respectively, measured using a BioTek FLX 80 spectrofluorophotometer and a Quant-iT PicoGreen dsDNA quantification kit (Invitrogen, Carlsbad, CA, United States) following the manufacturer’s instructions. PCRs were then ran with universal V4-targeting primers containing GeT-PlaGe adapters for the sequencing platform Genotoul^3^ (Forward : V4f_PlaGe 5′-CTT-TCC-CTA-CAC-GAC-GCT-CTT-CCG-ATC-TCC-AGC-A(C/G)C-(C/T)GC-GGT-AAT-TCC-3′, Reverse : V4f_PlaGe 5′-GGA-GTT-CAG-ACG-TGT-GCT-CTT-CCG-ATC-TAC-TTT-CGT-TCT-TGA-T(C/T) (A/G)-A-3′). Amplification was carried out in triplicate for each sample with the following PCR conditions: 98°C for 30 s, 12 cycles of 98°C for 10 s, 53°C for 30 s, 74°C for 30 s, 18 cycles of 98°C for 10 s, 48°C for 30 s, 74°C for 30 s and a final elongation step of 72°C for 10 min. Gel electrophoresis was used to assess the PCR amplifications. After pooling the triplicates, samples were purified using NucleoSpin Gel and PCR Clean-up (Macherey-Nagel, Hoerdt, France). Finally, purified products were diluted to obtain equimolar concentration before being sequenced by Illumina^®^ MISeq (2×250) at Genotoul. Samples tagging and library pools were performed at Genotoul. Quality check of the sequence data was performed via removal of reads with biased nucleotide (i.e., Phred score < 1%), incomplete or wrong primer sequence and chimaeras, via USEARCH (Edgar et al., 2011). After this cleaning step 1,064,259 distinct paired-end sequences remained, accounting for 9,562,085 reads, which accounted numbers on average to 39,842 reads per sample. In order to eliminate PCR errors and sample cross contaminations within the Illumina run, singletons, as well as reads present in less than two samples and having a total number of three over the whole data set, were removed. This rather strict selection brought to a 55% reduction of the dataset (from 9,562,085 to 472,810 distinct sequences). Run sequence data are available here upon request^4^. Before OTUs clustering, all sequences with percentage of identity (ID) to the reference database PR2 (Guillou et al., 2013) ≤80% were removed, considering that values under this threshold lead to unreliable taxonomic assignation (Stoeck et al., 2010; de Vargas et al., 2015; Mahé et al., 2017). The remaining reads were clustered by the agglomerative, unsupervised single-linkage-clustering algorithm Swarm 2 (Mahé et al., 2014, 2015), with a default clustering threshold of *d* = 1 (according to Mahé et al., 2015). OTU taxonomic assignation was performed comparing the representative read of each OTU to the V4 reference database. Metabarcodes matching the 18S rDNA sequences obtained by single cell with a similarity above 97% and showing abundance above 10 reads in at least one sample were selected for downstream analysis. This resulted in one barcode matching the sequences 10-044/10-045 (97.92% identity), five barcodes matching the sequence 13-374 (identity between 96.87% and 97.13%) and one barcode matching 12-150 (100% identity). The dynamics of these three groups were studied as a proxy for the presence of the subclades OOM_1_1 (seq. 10-045 related barcode), OOM_1_2 (seq. 13-374 related barcodes) and clade OOM_2 (12-150 related barcode), within the barcode dataset in relation to diatoms and *Pseudo-nitzschia* OTUs.

## Results

### Microscopic Observation of Endobiotic Parasitoids of *Pseudo-nitzschia* spp.

**FIGURE 1:**
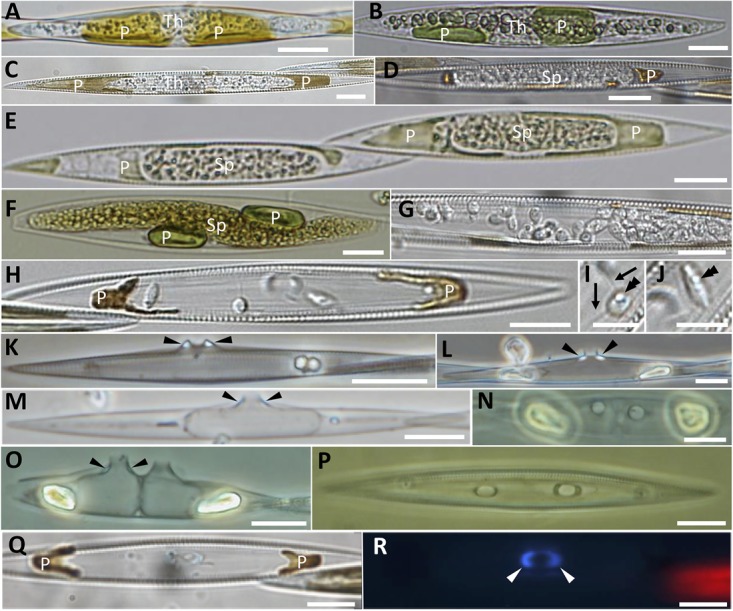
Morphological characterisation of the novel oomycete parasitoids of *Pseudo-nitzschia* described in this study. **(A–C)** Unwalled, increasingly granular thalli developing in *Pseudo-nitzschia australis* **(A,B)** and *Pseudo-nitzschia* cf. *pungens/multiseries* cells **(C)**; thalli are progressively pushing apart the degraded, yet still pigmented plastids. **(D–F)** Holocarpic syncytia maturing into sporangia, in *Pseudo-nitzschia* cf. *pungens/multiseries* **(D,E)** and *Pseudo-nitzschia australis* **(F)**. **(G–J)** Zoospore release in *Pseudo-nitzschia* cf. *pungens/multiseries*. Zoospores bear two flagella (arrows in **I**), a single refractive globule (double arrowheads in **I** and **J**) and their shape varies from almost spherical **(I)** to oblong **(J)**. **(K–R)** Empty sporangia. Arrowheads point to thickenings in the discharge tube. **(K)** *Pseudo-nitzschia pungens/multiseries*, valve view. **(L)** *Pseudo-nitzschia australis*, valve view. **(M)** Empty sporangium within *Pseudo-nitzschia fraudulenta*, valve view. **(N–P)** Two empty sporangia within a *Pseudo-nitzschia fraudulenta* frustule, girdle view **(N)** and valve view **(O)**, and *Pseudo-nitzschia pungens/multiseries*, girdle view **(P)**. **(Q,R)** *Pseudo-nitzschia* cf. *pungens/multiseries*, girdle view, under DIC **(Q)** and epifluorescence microscopy (**R**, Calcofluor-White staining), to highlight the thickening of the discharge tube. Scale bars = 10 μm except in **I**,**J** (5 μm). Samples in **(B,E,F)** were Lugol’s iodine fixed. **(B)** Refers to the sequenced cell *P. australis* parasitoid 10-044. Th, thallus; Sp, sporangium; *P*, plastid.

Endobiotic parasitoids infecting the diatoms *Pseudo-nitzschia* spp. and *Melosira* cf. *nummuloides* were observed in plankton samples collected in Brittany (France) and Scotland (United Kingdom) between 2010 and 2016. The endobiotic parasitoids infecting the planktonic pennate diatom *Pseudo-nitzschia* spp. share several morphological features (Figures 1A–R). The clearest observable stages of infection consist in swollen *Pseudo-nitzschia* cells symptomatic of the parasite thallus growing inside the diatom and pushing the valves apart. This stage has been observed in two different hosts; *Pseudo-nitzschia australis* (Figures 1A,B) and *Pseudo-nitzschia* cf. *pungens/multiseries* (Figure 1C). The hyaline, naked parasitoid thallus is at this stage difficult to visualise using light microscopy, and is most easily distinguished from the host cell thanks to the presence of several small refractive globules in the cytoplasm. The host plastids start collapsing early (Figures 1A,B) and their colour darkens from the healthy golden-yellow to brown (Figure 1C). Once the host is completely consumed, the parasitoid thallus condenses and adopts the shape of the future sporangium, as seen in *P.* cf. *pungens/multiseries* (Figures 1D,E) and *P. australis* (Figure 1F). At this stage, the cytoplasm of the parasitoid has a finely granular appearance, due to the increased number of small refractive globules and the rearrangement of the cytoplasm toward spore cleavage (Figures 1E,F). The dark brown remainders of the chloroplasts are pushed apart by the tip of the growing sporangium (Figure 1D), a morphologically distinctive feature often seen in later stages (Figures 1H–Q). Once the sporangium has formed, a rigid cell wall is deposited and the cytoplasm of the parasitoid is cleaved into spores that become flagellated (Figure 1G). These zoospores are motile within the sporangium, as observed in *P.* cf. *pungens/multiseries*. Once mature, they are directly discharged (see saprolegnoid discharge behaviour in Padgett and Johnson, 2004) outside the frustule via a broadly conical DT (Figure 1H). The zoospores are oblong to nearly spherical, each of them bearing a single refractive globule and two flagella (Figures 1I,J). The remaining empty sporangia are the most easily identifiable sign of the parasitoid infection (Figures 1K–R). Their overall structure is similar regardless the *Pseudo-nitzschia* host, i.e., a thin-walled sporangium filling almost completely the host frustule, and piercing it in the girdle bands region (cingulum) through a DT. So far, most of the morphological differences observed among the collected specimens concern the DT. Based on the shape of the mouth of the DT and the relative position of cell wall thickenings (also known as “forcing apparatus”), at least four different morphologies have been recorded in our observations. Specimens of infected *P.* cf. *pungens/multiseries* showed that the DT was oval to its exit, with the long axis 1.5–1.7 times its width (*n* = 3) and with extramatrical thickenings, forming a protruding beak-like structure (Figure 1K, arrowheads and 1P). The thickenings respond better to Calcofluor-White staining compared to the unstained sporangium cell wall (Figures 1Q,R, arrowheads). In three *P. australis* specimens where an empty sporangium could be observed, thickenings were located at the base of an oval DT (Figure 1L, arrowheads), itself protruding for ca. 2.5 μm outside the frustule. Two independent observations of sporangia from parasitoids infecting *P. fraudulenta* showed that the outline of the mouth of the DT was round (Figure 1N) with lateral, rather than basal, thickenings accompanying the 1.5–3 μm long DT (Figures 1M–O). Additionally, the sporangium did not adopt the shape of the host frustule, but instead developed its own sub-cylindrical/olpidioid shape. Finally, the observation of an empty sporangium of infected *P.* cf. *plurisecta* showed that the DT was more or less round with the long axis 1.2 times its width and with barely visible basal thickenings (not shown). Multiple sporangia within the same host cell with a DT developing from each sporangium were also observed in *P. fraudulenta* (Figures 1N,O) and *P. pungens/multiseries* (Figure 1P). From this sample, sexual reproduction and auxospores of *P. pungens/multiseries* were also observed at the same time as infected cells (not shown).

### Microscopic Observations of an Endobiotic Parasitoid of *Melosira* cf. *nummuloides*

**FIGURE 2:**
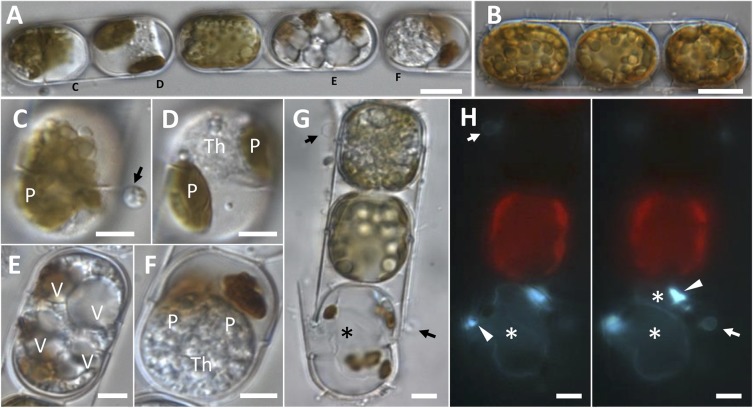
Morphological characterisation of a novel oomycete parasitoid of *Melosira* cf. *nummuloides.* **(A,B)**. Highly infected diatom chain **(A)**, compared to a healthy one **(B)**. **(C)** Early stage of infection, with a spore (arrow) bearing refractive globules, encysted on a diatom frustule. **(D)** Unwalled thallus developing within the host cell, the once numerous small plate-like plastids are collapsed in two olive-green masses. **(E)** Highly vacuolised parasitic thallus filling the host cell almost entirely. **(F)** Mature spherical multinucleate thallus filling most of the host cell, the disrupted plastids are represented by two brown masses. **(G,H)** Bright field **(G)** and epifluorescence (**H**, Calcofluor-White staining) microscopy of an uninfected cell (top) and a dead cell bearing one empty sporangium (bottom, asterisk) and an encysted spore (arrow). At a different focal plan (**H**, right panel), a second smaller parasitic sporangium was discernible (top asterisk). Sporangial wall thickenings in correspondence of the discharge tube were also visible (arrowheads). **(A,C–F)** Refers to the single infected colony sequenced to obtain *M*. cf. *nummuloides* parasitoid Melo1para sequence. P, plastids; V, vacuole; Th, thallus. Scale bars: 10 μm in **(A,B)**; 5 μm from **(C–H)**.

An endobiotic parasitoid of the centric benthic diatom *Melosira* cf. *nummuloides*, shared morphological characters with the parasitoids infecting *Pseudo-nitzschia* spp., (Figures 2A–H). Heavily infected clonal colonies showed different stages of development (Figure 2A), and could easily be distinguished from healthy cells (Figure 2B). Settled spores could be observed on the host frustule (arrowed in Figures 2C–H); they were 3 μm in diameter, with refractive globules visible in light microscopy (Figure 2C, arrow) and a Calcofluor-White positive cell wall (Figure 2H, arrows). As in *Pseudo-nitzschia* spp. the parasitoid starts developing inside the host cell as an unwalled cytoplasmic thallus (Figure 2D) and evolves into a coarsely granular cytoplasmic mass with many submicrometric refractive globules (Figure 2G, top cell); the light brown, plate-like plastids of *Melosira* quickly condense into few masses of dark brown material (Figures 2A–G) and lose chlorophyll autofluorescence (Figure 2H). The highly vacuolated thallus (Figure 2E) and the spherical body containing a dense granular cytoplasm (Figure 2F) were not observed in infected *Pseudo-nitzschia* spp. cells. Two saccate empty sporangia were seen to fill almost completely the frustules of the host diatom (Figure 2H asterisks), each with a DT breaking through the cingulum (Figures 2G,H, left). Cell wall thickenings were also visible, but their position relative to the DT was found to be variable (sometimes completely dissociated from the DT) and could not be unambiguously established (Figure 2H, arrowheads).

### Phylogeny of the Two Novel Clades of Oomycete Parasitoids

**FIGURE 3:**
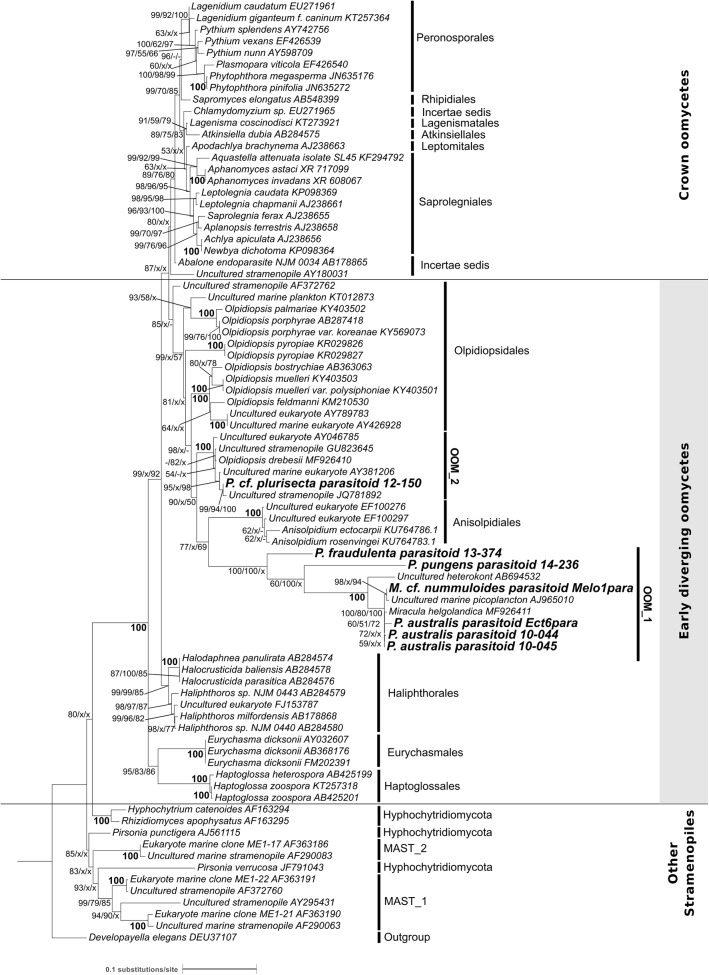
Maximum likelihood phylogenetic tree reconstruction based on partial 18S rDNA genes of novel diatom parasitoids within the Oomycota (Stramenopila), inferred from 81 sequences on 1,206 positions. New sequences are in bold and they are named according to the host species and the sample ID within the new clades OOM_1 and OOM_2 (details in Table 1). Bootstrap values (1,000 replicates for ML and NJ, 100 for MP) are shown at each node as a percentage for the three computational methods tested, with the following order: maximum likelihood, neighbour joining and maximum parsimony. X, node not supported; –, bootstrap support <50%. Bold numbers indicate agreement on the same bootstrap value for the three methods. Refer to the alignment in Supplementary Data Sheet S2.

**FIGURE 4:**
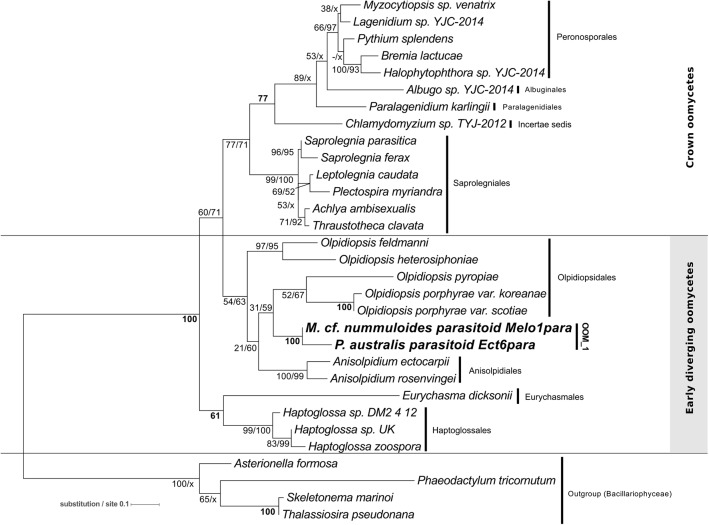
Maximum-likelihood phylogenetic tree reconstruction using 31 concatenated amino acid sequences for the mitochondrial genetic markers *cox1* (194 positions) and *cox2* (185 positions) of the parasitoid of *Melosira* cf. *nummuloides* (in bold) within the oomycetes (Stramenopila). The *cox2* amino acid sequence of the parasitoid of *P. australis* Ect6para (in bold) was also included. Four diatoms (Stramenopila, Bacillariophyceae) were defined as outgroup. Bootstrap values (1,000 replicates) are shown at each node for ML and NJ methods, respectively. Bold numbers indicate agreement on the same bootstrap value for both methods. Refer to the alignment in Supplementary Data Sheet S3.

The phylogeny of the parasitoids was reconstructed using 18S rDNA and *cox1* and *cox2* sequences obtained from the single diatom cells detailed in Table 1 and Supplementary Figure S5. In NJ, ML and MP topologies, computed with (Figure 3) or without Gblocks trimming (Supplementary Figure S1), all 18S rDNA sequences of our diatom parasitoids cluster into a clade that contains oomycete parasites of brown and red macroalgae, the Anisolpidiales and Olpidiopsidales (Figure 3). This branch (99% in ML and 57% in MP, not supported in NJ) forms, together with the Eurychasmales, Haptoglossales, and Haliphthorales, a group of mostly marine oomycetes, called “early diverging Oomycetes” (*sensu* Beakes et al., 2012). This group is opposed to the well-studied, mainly terrestrial or freshwater pathogen and saprobe lineages of oomycetes composed of the Saprolegniales, Leptomitales, Atkinsiellales, Peronosporales, Rhipidiales, Lagenismatales, and other unclassified oomycetes, known as “crown oomycetes.” Our seven new sequences of diatoms parasitoids further separate into two well-supported clades, that we named here OOM_1 (OOM:Oomycetes) and OOM_2, designed in compliance with the EukRef guidelines for defining environmental clades (Berney et al., 2017). The clade OOM_1 is sister to the order Anisolpidiales (*sensu* Gachon et al., 2017); it includes *M. helgolandica* (a parasitoid of *P. pungens*, Buaya et al., 2017) and the parasitoids of *P. australis* (three sequences), *P. pungens* and *P. fraudulenta* as well as one parasitoid of *Melosira* cf. *nummuloides* (bootstrap value 100% with all clustering methods). Within OOM_1, one sub-clade, again supported at 100% in NJ, ML and MP, contains the parasitoids of *P. australis* and *M*. cf. *nummuloides*, *M. helgolandica* and two uncultured organisms. OOM_1_1 is sister to *P. pungens* parasitoid in ML and NJ with 60% and 100% bootstrap support, and this group is sister to *P. fraudulenta* parasitoid with 100% bootstrap in all methods except MP, which fails to retrieve the same topology. The parasitoid isolated from *P.* cf. *plurisecta* falls into the highly supported clade OOM_2 (100% ML, NJ and MP). This clade is sister to the Anisolpidiales and OOM_1, and includes four environmental sequences previously annotated as Stramenopiles *incertae sedis*, as well as the parasitoid from *R. imbricata*, *O. drebesii*. Though less informative than the 18S tree, ML and NJ topologies obtained with the concatenated mitochondrial markers *cox1* and *cox2* also confirm the placement of the *M*. cf. *nummuloides* parasitoid Melo1Para and *P. australis* parasitoid Ect6para within a strongly supported cluster (100% bootstrap with both methods), despite the availability of *cox2* only for the latter parasitoid. The two parasitoids are placed within a weakly supported (54 and 63%, for ML and NJ) [*Olpidiopsis* and *Anisolpidium*] clade (Figure 4).

### Global Distribution, Diversity, and Local Ecological Patterns

**FIGURE 5:**
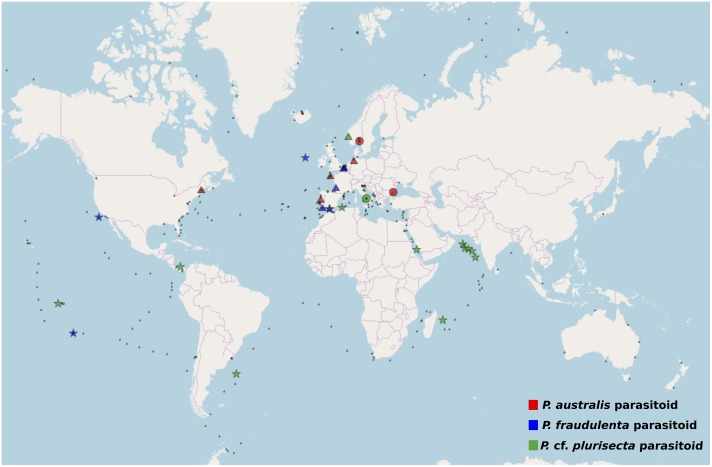
Worldwide screening for the presence of *Pseudo-nitzschia* oomycete parasitoid barcodes. The metabarcode databases Ocean Sampling Day (triangle), BioMarKs (circle), and TARA (star) were searched for the presence of >10 paired reads over 99% identical to the V4 or V9 region of the 18S rDNA of the parasitoids *P*. cf. *plurisecta* 12-150 (green) within OOM_2; *P. fraudulenta* 13-374 (blue), *P. australis* 10-044/10-045 (red) within OOM_1. Small black symbols show the absence of reads matching the searched organisms in the relative database.

**FIGURE 6:**
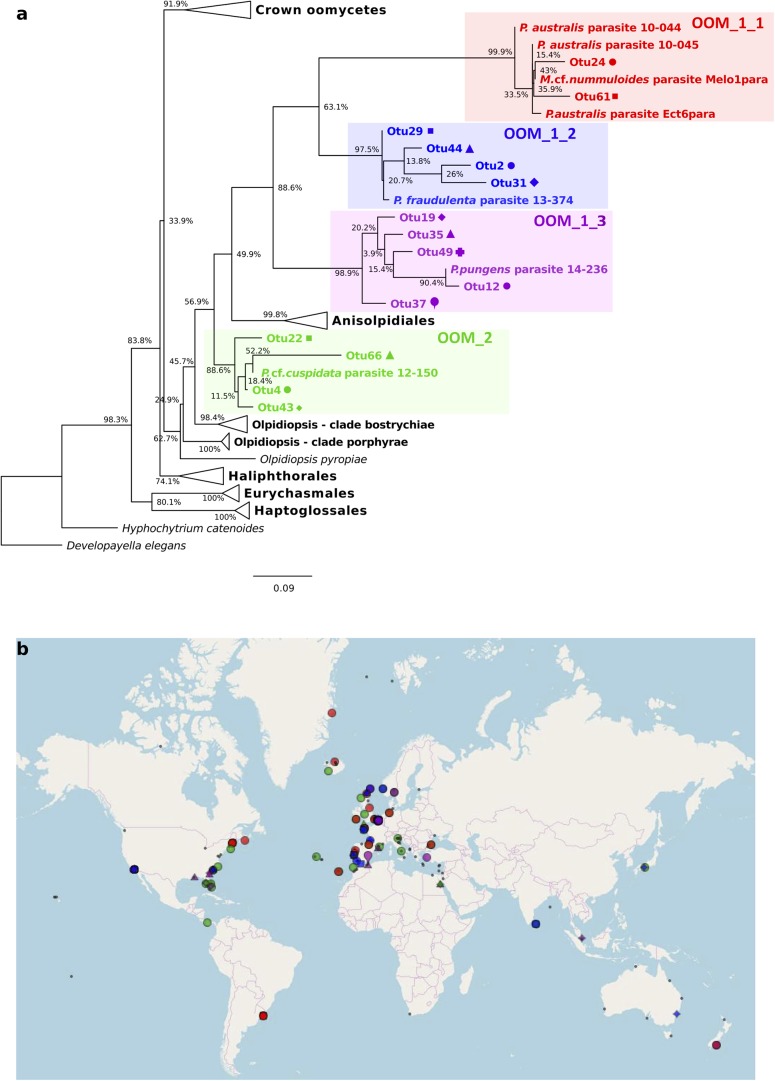
Diversity and distribution of OOM_1 and OOM_2 related OTUs based on public SRA marine barcoding datasets. The colour code identifies each one of the four new clades of diatom parasitoids OOM_1_1 (red), OOM_1_2 (blue), OOM_1_3 (purple) and OOM_2 (green). **(a)** Maximum Likelihood tree of 15 18S rDNA V4 OTUs retrieved by the MOULINETTE Pipeline. Refer to the alignment in Supplementary Data Sheet S4. **(b)** Global distribution of each detected OTU based on GPS coordinates of matching SRA runs. Within-clade symbols are attributed to different OTUs.

**FIGURE 7:**
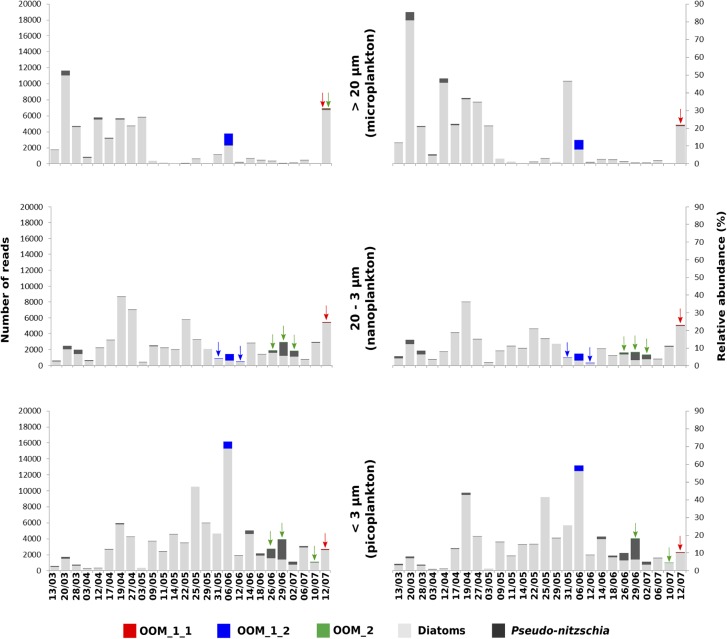
Phenology of the parasitoid of *P.* cf. *plurisecta* 12-150 (OOM_2, green) and of putative parasitoids within the sub-clades OOM_1_1 (red) and OOM_1_2 (blue), in relation to the diatom community during the spring/summer 2012 in the Bay of Concarneau (France), described by metabarcoding survey. The three left hand-side histograms show the total abundance of reads on the *y*-axis, whilst the ones on the right hand side show the relative abundances as a percentage of the total eukaryotic community. The size fractions for microplankton (top, >20 μm), nanoplankton (middle, 20–3 μm) and picoplankton (bottom, 3–0.2 μm) are indicated in the middle. *Pseudo-nitzschia* OTUs are in dark grey, other diatom OTUs in light grey. Sampling dates are shown on the bottom *x*-axis.

The V4 and V9 hypervariable regions of the 18S rDNA sequences obtained from our SC isolates have been used to perform a stringent screening of public databases. Reads matching our reference sequences of parasitoids of *P. fraudulenta, P. australis* and *P.* cf. *plurisecta* were found in diverse marine zones, such as semi-enclosed, temperate high salinity regions (Mediterranean and Red Sea) and the three major oceans (Indian, Atlantic and Pacific, Figure 5). In publicly available databases, reads matching the parasitoid of *P. australis* 10-044/10-045 (red) within the OOM_1 clade were not found in the Mediterranean Sea, but in the Black Sea. In the Indian Ocean, only reads matching the parasitoid of *P*. cf. *plurisecta* 12-150 (green) within the OOM_2 clade were found. Using all our Sanger sequences as queries (Table 1) of public datasets, we identified 11 and 4 additional OTUs that fall within OOM_1 and OOM_2, respectively. Within OOM1, two OTUs fall into the subclade defined by the sequences of the *P. australis* and *M.* cf. *nummuloides* parasitoids, with 99.9% bootstrap support; four OTUs group with the sequence of the *P. fraudulenta* parasitoid, and define a sub-clade supported with 97.5% bootstrap; five OTUs cluster with the parasitoid of *P. pungens* and form another sub-clade supported with 98.9% bootstrap. Therefore, we named these three well-supported subclades as OOM_1_1, OOM_1_2, and OOM_1_3, respectively (Figure 6A). These 15 OTUs further highlight a wide geographic distribution of the taxa within the OOM_1 and OOM_2 clades, at a global scale (Figure 6B). Bearing in mind that the type species for the genus of diatom parasitoids *Ectrogella* was first described infecting a freshwater diatom species of *Synedra* (Zopf, 1884), we attempted to detect sequences related to OOM_1 and OOM_2 in a custom selection of 19,000 public freshwater metagenomic datasets. Some sequences of soil- and freshwater-dwelling oomycetes were also included as a positive control. As expected, this screen yielded OTUs closely related to “crown” oomycetes (e.g., *Aphanomyces*, *Saprolegnia*, *Phytophthora*) and early-diverging terrestrial oomycetes (e.g., *Haptoglossa*). However, none of the OTUs retrieved from these non-marine environments could be confidently assigned to OOM_1 or OOM_2 clades as per their best blast hit against the n/r Database and 18S phylogenetic reconstruction (not shown). From March to July 2012, during the DYNAPSE surveys in the Concarneau Bay (Brittany, France, 47° 49′ N 3° 56′ W), 12 OTUs annotated as oomycetes were identified by a metabarcoding approach of different size-fractioned protists communities. Single metabarcodes matching the parasitoid of *P. australis* (10-044/10-045, 97.9% identity) and the parasitoid of *P.* cf. *plurisecta* (12-150, 100% identity), respectively, and five metabarcodes matching the parasitoid of *P. fraudulenta* (13-374, 96.8% to 97.1% identity) were found. These metabarcodes were used as proxies to investigate the dynamics of the subclades OOM_1_1 (related to the parasitoid of *P. australis* 10-044/10-045), OOM_1_2 (related to the parasitoid of *P. fraudulenta* 13-374) and the clade OOM_2 (related to the parasitoid of *P.* cf. *plurisecta* 12-150) (Figure 6a), in particular in relation to diatoms and *Pseudo-nitzschia* OTUs (Figure 7). Ephemeral increases in read abundance were detected for all the three investigated oomycetes, that were otherwise absent or below the threshold value of 10 reads per sample in the rest of the time series. The highest recorded number of reads for the investigated parasitoid was retrieved on the 6th of June with 1,500 reads assigned to OOM_1_2 in the microplanktonic (>20 μm size-fraction), followed by the 832 in the nanoplankton (3–20 μm size-fraction) and 834 in the picoplankton (0.2–3 μm size-fraction) (5.23%, 3.97%, and 3.05%, respectively, in relative abundances). The peak of the 6th of June was preceded (31st of May) and followed (12th of June) by the detection of reads belonging to OOM_1_2 in the nanoplankton fraction only. This peak in OOM_1_2 abundance co-occurred with an early-summer diatom bloom. On June the 6th, diatom OTUs represented 8% (microplankton), 2.8% (nanoplankton) and 56.1% (picoplankton) and *Cerataulina pelagica* OTU accounted for 7.48% (microplankton), 2.54% (nanoplankton) and 51.68% (picoplankton) whilst *Pseudo-nitzschia* OTUs were below the 10 reads threshold (Supplementary Figure S2). Reads associated to the parasitoid of *P.* cf. *plurisecta* in clade OOM_2 appeared at low abundances in the samples from the 26th and 29th of June and 2nd of July, in the nanoplankton, with read numbers of 27, 27 and 11, respectively (0.10%, 0.06%, 0.03% relative abundances). On the 29th of June low presence for this metabarcode was also detected in the picoplankton (11 reads, 0.05% in relative abundance), as it was on the 10th of July (25 reads, 0.10% relative abundance). The highest abundance was recorded on the last day of the time series (12th of July) and was 31 reads, 0.09% of the microeukaryotic community. This co-occurred with the third increase in diatom presence in the time series, the first being the spring bloom (20th of March – 5th of May) when none of the diatom parasitoid barcodes was significantly abundant (Supplementary Figure S3). From June the 14th diatoms increased, especially in the two smaller size fractions. On this occasion *Pseudo-nitzschia* OTUs were well represented, reaching abundances as high as 11.59% of the picoeukaryotes (4.3% of the nanoplankton and 0.18% of the microplankton) on the 29th of June. Microscopy sample observations (data not shown) revealed a higher abundance of cells belonging to the *P. delicatissima* group (i.e., *Pseudo-nitzschia* cells with transapical axis < 3 μm in valve view; this group encompasses *P. plurisecta*) as compared to cells belonging to the *P. seriata* group (transapical axis > 3 μm in valve view, encompassing *P. australis, P. fraudulenta* and *P. pungens/multiseries*) during these dates. Finally the barcode within OOM_1_1 appeared only once at low abundances in the last date of the time series (July 12^t^h) with 27, 13, and 15 reads (accounting for 0.08%, 0.05%, and 0.05% of the microeukaryotic community) in the micro, nano and picoplankton, respectively, (Supplementary Figure S4). On the same day an increase of an OTU assigned to the diatom family Mediophyceae was also observed (12.97%, 12.84%, and 4.99% of the total eukaryotic community in the micro, nano and picoplankton, respectively, data not shown). Monitoring of environmental conditions showed that salinity was stable during the sampling mission (34.8 on average). The surface water temperature ranged from a minimum of 10.14°C to a maximum of 17.23°C during the period monitored. The studied organisms were detected at water temperatures ranging from 13.77°C to 17.23°C (data not shown).

## Discussion

### Taxonomic Affiliation of the New Diatom Parasitoids

**FIGURE 8:**
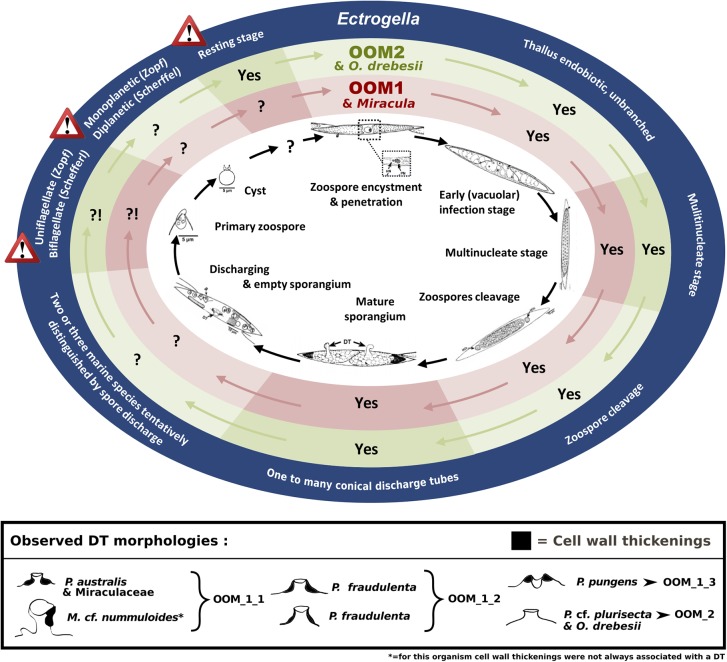
Mapping observed OOM_1 and *Miracula helgolandica* (Buaya et al., 2017) and OOM_2 and *Olpidiopsis drebesii* (Buaya et al., 2017) life stages against *Pseudo-nitzschia pungens* oomycete parasitoid life cycle (Hanic et al., 2009) and the defining morphological criteria for the *Ectrogella* genus (Zopf, 1884; Scherffel, 1925). The development cycle of the oomycete parasitoid of *P. pungens* described by Hanic et al. (2009) is reproduced in the inner ellipse. The key morphological criteria defining the genus *Ectrogella* Zopf emend. Scherffel are highlighted in the external dark blue ellipse. The warning signs highlight contradictions between the original descriptions of the type species given by Zopf and Scherffel. The intercalary red and green ellipses highlight the congruence of each criterion (Yes) or lack thereof (?!, when morphology unknown or conflicting) between these original *Ectrogella* descriptions and the morphology of the OOM_1 and OOM_2 clades, respectively. The box at the bottom summarises the different DT morphologies as observed in different diatom hosts. Black colour highlights the cell wall thickenings (“forcing apparatus” *sensu* Sparrow, 1960).

Among all known holocarpic pathogens of diatoms, OOM_1 and OOM_2 are clearly distinct from *Lagenisma coscinodisci*, both morphologically and molecularly (Thines et al., 2015). *Aphanomycopsis bacillariacearum* and *Lagenidium enecans* are two lesser known parasitoids of diatoms that still remain to be characterised molecularly (Scherffel, 1925). However, the combined absence in our observations of branched sporangium, persistent infection tube, elongated DT, primary spore encysting at the mouth of the DT, and of thick-walled resting spore make an affiliation of OOM_1 or OOM_2 to either of these taxa most unlikely. Therefore, the only known taxa to which OOM_1 and OOM_2 might correspond are either *Ectrogella*, *Olpidiopsis*, *Anisolpidium* or *Miracula*. This hypothesis will be discussed in the following paragraphs. The morphological and developmental features of the parasitoids observed during this study suggest they most closely match the genus *Ectrogella*, and also closely resemble the holocarpic parasitoid observed on *P. pungens* by Hanic et al. (2009) (Figure 8). However, like these authors, we could not match any of these parasitoids to an already described species of *Ectrogella*, because species delimitation in this genus is based on much-debated criteria relating to resting spores, which were not observed here (Figure 8). More precisely, the genus *Ectrogella* was originally described in freshwater diatoms (*Synedra* spp.) on the basis of an intramatrical, unbranched and holocarpic thallus, producing uniflagellate, diplanetic spores (Zopf, 1884). Scherffel subsequently gave a conflicting account of spore flagellation and generations (Scherffel, 1925). These initial descriptions were followed by numerous, sometimes patchy, observations that have aggregated more criteria, such as the presence of sexual reproduction or resting spores. In his synthesis, Sparrow (1960) recognised eight species of *Ectrogella*; seven of them infecting diatoms, with only three in marine hosts: *E. licmophorae* (characterised by spores encysting at the mouth of the sporangium, i.e., achlyoid), *E. perforans* (direct spore discharge, i.e., saprolegnoid) and *E. eurychasmoides* (primary spores encysting within the sporangium, i.e., eurychasmoid). Most recently, all the freshwater species of *Ectrogella* were synonymised to *E. bacillariacearum* (with the exception of *E. monostoma*, synonymised with *Aphanomycopsis bacillariacearum*) and the number of extant marine species was reduced to two, by including *E. licmophorae* into *E. perforans*, and keeping *E. eurychasmoides* as a “doubtfully distinct” species (Dick, 2001). Overall, this much debated taxonomy is based on conflicting accounts of spore morphology (uni- or biflagellate) and debatable spore discharge behaviour (Johnson, 1966; Padgett and Johnson, 2004). We were largely unable to observe any of these criteria, except for the parasitoid of *P.* cf. *pungens*/*multiseries*. Additionally, the clear polyphyly of the parasitoids observed here casts serious doubt as to whether all species that have tentatively been synonymised with extant *Ectrogella* taxa really correspond to identical, or even related, species. Finally, bearing in mind that the type species for *Ectrogella* (*E. bacillariacearum*) was described in a freshwater diatom (*Synedra* sp.), a screening of 19,000 metabarcoding datasets from freshwater environments was conducted (totalling 487 Gb of 18S amplicons). Despite *Synedra* being present, no sequence affiliated to either OOM_1 or OOM_2 was retrieved, when in comparison, 15 OTUS affiliated to OOM_1 and OOM_2 were retrieved from a smaller marine dataset (totalling 43 Gb of 18S amplicons). Therefore, a hypothesis that should be fully considered is that OOM_1 and OOM_2 may be exclusively marine and that the type species for *Ectrogella* might be distinct from both clades. For all these reasons, the genus name *Ectrogella* cannot be confidently assigned to either OOM_1 or OOM_2 (or a subclade thereof). Phylogenetically, OOM_1 and OOM_2 are most closely related to *Anisolpidium*, and to marine species of the genus *Olpidiopsis*. The genus *Anisolpidium* was recently reassigned to the Oomycota and encompasses endobiotic, holocarpic pathogens of brown algae (Gachon et al., 2017). Recent evidence obtained on *Olpidiopsis* - all molecularly characterised species of which are endobiotic, holocarpic pathogens of marine red algae - suggests that this genus is possibly polyphyletic (Badis et al. *subm.*). In the light of their different algal hosts and their well-supported molecular divergence, we conclude that a distinction should be established between the clades OOM_1, OOM_2, and the genera *Anisolpidium* and *Olpidiopsis*. Our data, however, do not allow us to decide if the clades OOM_1 and OOM_2 correspond to any of the two genera, or if they should be separated at a higher taxonomical rank. Subsequently, the sub-clades OOM_1_1, OOM_1_2, OOM_1_3 as well as OOM_2 could correspond to four species spanning two genera, or maybe correspond to taxa of higher rank. For the time being, awaiting morphological corroborations, life cycle analyses, new sequence data, and a taxonomic revision which resolves the exact relationships between the orders Anisolpidiales and Olpidiopsidales, a taxonomic rank to the OOM_1 and OOM_2 clades is not assigned to avoid the risk of describing unstable species and genera. The Miraculaceae - the only known member of which falls within OOM_1_1 (100% bootstrap with all methods) - were described recently as a novel oomycete family sister to all other oomycete clades characterised to date, solely on the basis of a long branch in an 18S rDNA phylogenetic reconstruction (Buaya et al*, 2017*). The morphology of the DT was proposed as the only morphological synapomorphy defining the family and within it, its type genus and type species. Our parasitoids of *P. australis* matched the described DT morphology for *Miracula* (Figure 1L), and both cluster into OOM_1_1. However, the parasitoid of *Melosira* cf. *nummuloides*, which also falls into OOM_1_1 had conflicting DT morphology (Figures 2B,H). This variation of DT morphology that we observed, not only within OOM_1_1, but also within OOM_1, unavoidably calls for a morphological re-description of the Miraculaceae, the genus *Miracula*, and perhaps the species *M. helgolandica*, in order to accommodate our observations. Giving the morphological variability of the DT within OOM_1 and its subclades, however, the validity of using DT morphology to support the delineation of taxa within OOM_1 is questionable. In fact, this was already highlighted when distinct sporangium morphologies, number and shapes of DTs and spore release methods were observed in a single infected population of *Licmophora* sp. (Johnson, 1966). This author even highlighted variations of the cell wall thickenings across several DTs of the same sporangium, further suggesting that this criterion is taxonomically unreliable. Our observations of the *M*. cf. *nummuloides* and *Pseudo-nitzschia* spp. parasitoids confirm that sporangia can greatly vary in shape despite a close molecular relatedness (i.e., within the sub-clade OOM_1_1) or appear similar across more distantly related groups (i.e., between OOM_1 and OOM_2). In light of this, we consider the taxa Miraculaceae, *Miracula* and *M. helgolandica* unstable and await additional morphological or ultrastructural data to suggest a meaningful species/genus separation within OOM_1. This is why, having carefully considered all possibilities and failed to confidently assign this novel clade (or a sub-clade thereof) to any taxon, we elect to designate it according to a temporary nomenclature compliant with the UniEuk guidelines for naming environmental sequences (Berney et al., 2017). In conclusion, the molecular data collected here, which include longer 18S rDNA sequences, a much improved representation of *Olpidiopsis* diversity and the inclusion of the genus *Anisolpidium* (Gachon et al., 2017; Badis et al. *subm*.), as well as the mitochondrial markers *cox1* and *cox2*, all agree that *Miracula helgolandica* in fact belongs to the OOM_1 clade. Therefore, the rationale that underpinned the creation of the novel family Miraculaceae on the basis of a moderately supported clade sister to all the remaining oomycetes needs to be reconsidered and the boundaries of the genus *Miracula* within OOM_1 remain to be determined. Within OOM_1, our topology places *P. pungens* parasitoid (14-236) as sister to the group of parasitoids of *P. australis*/*M*. cf. *nummuloides* (Figure 3) and leaves the parasitoid of *P. fraudulenta* 13-374 sister to the rest of OOM_1. Molecular data obtained from sample 14-236 contained a lesser amount of phylogenetic information, due to 64 unassigned bases and an overall shorter aligned sequence (456 bp). The positions of the parasitoids of *P. fraudulenta* and *P. pungens* relative to one another or to the parasitoids of *P. australis*/*M*. cf. *nummuloides*, is therefore difficult to ascertain. Clade OOM_1_1, however, contains three sequences of parasitoids infecting *P. australis* and one infecting *M.* cf. *nummuloides.* The sequence identity between the parasitoid of *M*. cf. *nummuloides* and the parasitoids of *P. australis* exceeds 98.6% over 944 bp within the 18S rDNA (97.3% for the 422 bp of the V4 hypervariable region), similar to the identity level within the *P. australis* parasitoids group (above 98.7% on 944 bp and above 98.3% on the V4 hypervariable region). Typical thresholds used for species delimitation based on molecular information vary between 97% and 99% identity, though there is no widely accepted consensus threshold for either of the genetic markers used in this study (18S rDNA, *cox1*, and *cox2*, Caron et al., 2009; Beakes et al., 2012; Choi et al., 2015). Hence, we cannot exclude that the OOM_1_1 parasitoids are conspecific, despite infecting different hosts. This potential lack of host specificity further strengthens our choice to not assign a taxonomic rank (e.g., genus or species) to OOM_1_1, and to not proceed with its formal description. A second implication of this potential lack of host specificity is that it would be speculative to match the ultrastructural data acquired by Hanic et al. (2009) on a parasitoid of *P. pungens* with either OOM_1_1 (as described by Buaya et al., 2017), OOM_1_3 (our data, Figure 6a) or perhaps even any other of our novel (sub)clade(s). OOM_2 forms a discrete and strongly supported clade sister to the [OOM_1 and *Anisolpidium*] group (90% in ML, 50% in MP, unsupported in NJ), that contains the parasitoid isolated from *P.* cf. *plurisecta* together with two uncultured eukaryotes sequenced in the context of marine stramenopiles diversity surveys (Massana et al., 2004; Lin et al., 2012), and the recently defined *Olpidiopsis drebesii* (Buaya et al., 2017). According to the extensive dataset developed here, OOM_2 is only weakly related to other *Olpidiopsis* species, despite it containing *Olpidiopsis drebesii* (Buaya et al., 2017). Additionally the genus *Olpidiopsis* is widely reckoned as para- or polyphyletic and in need of extensive revision (Beakes et al., 2014; Beakes and Thines, 2016; Badis et al. *subm.*), therefore any taxonomical treatment of OOM_2 would at best be hypothetical.

### Host Preference, Ecology, and Distribution of the New Parasitoids

Although the sampling in this study focussed on the toxigenic genus *Pseudo-nitzschia*, convergent lines of evidence suggest that at least some of the OOM_1 and OOM_2 taxa are not strictly host specific. As stated above, parasitoids in sub-clade OOM_1_1 were isolated on two different hosts, i.e., *Pseudo-nitzschia australis* and *Melosira* cf. *nummuloides*. These two diatoms are both coastal and euryhaline, but fairly distinct in terms of phylogeny and habitat, the former being a raphid pennate planktonic diatom whilst the latter is a benthic centric diatom. Furthermore *M. helgolandica*, also belonging to OOM_1_1, has been isolated on a different *Pseudo-nitzschia* species (Buaya et al., 2017). Even if our molecular data are insufficient to decide whether the organisms infecting *Melosira*, *P. australis*, and *P. pungens* are conspecific, the possibility that the clade OOM_1_1 coincides with a single generalist or heteroxenous parasitoid should be considered. The same case applies, within OOM_2, to *O. drebesii*, isolated from the centric planktonic diatom *Rhizosolenia imbricata* (Buaya et al., 2017) despite a high molecular relatedness with the parasitoid of *P.* cf. *plurisecta*, yet again advocating the idea of a generalist parasitoid. Although the metabarcodes belonging to OOM_1 and OOM_2 were detected in the context of molecular environmental sampling, there is no indication that OOM_1 and OOM_2 would potentially infect hosts other than diatoms. The highest abundance of parasitoid reads was recorded on June the 6th and belonged to the subclade OOM_1_2, (ca. 97% similar to the V4 18S rDNA of *P. fraudulenta* parasitoid). The most likely host of this putative parasitoid was the centric planktonic diatom *Cerataulina pelagica*, which accounted for 92.14% of all diatom reads, thus forming a nearly monospecific bloom. On that date, the abundance of *Pseudo-nitzschia* was very low compared to the parasitoid read abundance (Supplementary Figure S2), and direct cell count confirmed this finding (data not shown). Furthermore, while a crash in *C. pelagica* abundance coincided simultaneously to a peak in OOM_1_2, the abundance of *Pseudo-nitzschia* OTUs grew steadily until June the 29th, to reach the highest abundance in the dataset, further supporting the idea of *Cerataulina* as OOM_1_2 host. The co-occurrence of a barcode 100% identical to the V4 18S rDNA region of the parasitoid infecting *P.* cf. *plurisecta* (OOM_2) with a bloom of *Pseudo-nitzschia* species belonging to the *P. delicatissima* group may suggest that in this case the oomycete was infecting either the same or a closely related diatom host. The same OOM_2 barcode was also co-occurring with an OOM_1_1 related barcode (97.9% similar to the V4 18S rDNA of *P. australis* parasitoids 10-044/10-045) on the 12th of July, when *Pseudo-nitzschia* were virtually absent, whilst OTUs related to an unknown Mediophyceae were increasing (data not shown). These results can therefore suggest heteroxeny for the OOM_2 parasitoid isolated from *P*. cf. *plurisecta* and possibly increase the putative hosts list for the OOM_1_1 subclade. Unfortunately microscopic observations of diatom infection could not be attempted within the context of the DYNAPSE metabarcoding survey for any of the detected OOM barcodes, since this mission was not specifically conceived to study planktonic parasitism. Weak read numbers for both OOM metabarcodes and their co-occurrence with multiple diatom species complicates the interpretation of the host specificity and temporal dynamics of our parasitoids. Our metabarcoding data suggest that the presence of these organisms is ephemeral, with occurrences that never last more than 1 week at most (Figure 7 and Supplementary Figures S2–S4). The relatively small read numbers retrieved for our parasites might be due to: (i) undersampling of the planktonic diversity caused by the filtration procedure and/or an unsaturating sequencing depth (Smith and Peay, 2014), (ii) a very short life cycle and a limited persistence in the water column, difficult to capture with our sampling frequency, (iii) a genuine rarity in the environment. Even if it is hard to disentangle the role of these three factors on the basis of this first survey, these data will help designing field missions targeting the ecology and host spectrum of OOM parasitoids. Moving from a regional to a global perspective, this analysis suggests a broad distribution of the sequenced parasitoids, with occurrences of reads spanning water bodies as diverse as the Atlantic, Indian and Pacific Oceans, the Mediterranean, Black, Red, North and Caribbean Seas (Figure 5). This widespread distribution is even broader when OTUs (>97%) related to OOM sequences are considered (Figure 6b), although the metabarcoding datasets analysed are not time series and accordingly, do not allow the inference of clear biogeographical patterns. A high sampling frequency will be required to resolve the seasonal and biogeographical patterns of such dynamic – putatively rare – protists that interact with other organisms. Therefore, the ecological relevance of these organisms needs to be addressed through directly targeted plankton surveys. In parallel, the establishment of laboratory cultures is paramount to investigate the life cycle and physiological traits of OOM parasitoids, which will underpin the understanding of their role and importance in plankton dynamics.

## Data Availability

All sequences obtained from single cell isolates are available in GenBank under the accession numbers specified in Table 1. The complete DYNAPSE metabarcoding dataset is currently available from the authors upon request and will be made available through a dedicated and stable repository.

## Author Contributions

RS, CG, and EB conceived the study. AG, EN, and GB carried out the sampling and molecular work. AG, YB, and PA carried out the data analysis. AG and CG wrote the manuscript, with contributions from all co-authors.

## Conflict of Interest Statement

The authors declare that the research was conducted in the absence of any commercial or financial relationships that could be construed as a potential conflict of interest.

## Footnotes

1 http://taraoceans.sb-roscoff.fr/EukDiv/

2 http://www.gpsvisualizer.com/

3 http://get.genotoul.fr/

4 sextant.ifremer.fr/record/16bc16ef-588a-47e2-803e-03b4acb85dca/

## Abbreviations

DT: discharge tube
ML: maximum likelihood
MP: maximum parsimony
NJ: neighbour joining.
