# Supplementary material for: Novel Widespread Marine Oomycetes Parasitising Diatoms, Including the Toxic Genus Pseudo-nitzschia: Genetic, Morphological, and Ecological Characterisation
Source: Front Microbiol. 2018 Dec 3;9:2918. doi: 10.3389/fmicb.2018.02918 (PMC6286980; doi:10.3389/fmicb.2018.02918)
Supplement: Supplementary file 1 [file Table_1.pdf]

Primer name	Reference	Sequence 3'-5'
18SFW	Grzebyk *et al.*, 1998	TCCTGCCAGTAGTCATATGC
18SRV	Grzebyk *et al.*, 1998	TGATCCTTCGGCAGGTTCAC
D3B	Nunn *et al.*, 1996	TCGGAGGGAACCAGCTACTA
F139	Gachon *et al.*, 2017	AGTCTATTTGATAGTACCTTACTAC
R1233	Gachon *et al.*, 2017	CAATCCTTACTATGTCTGG
18R7	Chomérat *et al.*, 2010	TGGAGCTGGAATTACCGCGGCT
1250R	Hoppenrath *et al.*, 2007	TAACGGAATTAACCAGACA
SR9PR	Yamaguchi and Horiguchi, 2005	AACTAAGAACRGCCATGCAC
SR9FW	Freeman *et al.*, 2004	TGCGGCTTAATTTGACTCAAC
1050F	Chantangsi and Leander, 2010	GGGGGAGTATGGTCGCRAG
28SKARREV	Nézan *et al.*, 2012	AAACGGGAYTCTCACCCTC

Table S1: Primers used in this study

References

Chantangsi, C. and Leander, B.S. (2010) An SSU rDNA barcoding approach to the diversity of marine interstitial cercozoans , including descriptions of four novel genera and nine novel species. *Int. J. Syst. Evol. Microbiol.* **60**: 1962–1977.

Chomérat, N., Sellos, D.Y., Zentz, F., and Nézan, E. (2010) Morphology and molecular phylogeny of prorocentrum consutum sp . nov . (dinophyceae), a new benthic dinoflagellate from south Brittany (northwestern France). *J. Phycol.* **46**: 183–194.

Freeman, M., Hiroshi, Y., and Kazuo, O. (2004) A microsporidian parasite of the genus Spraguea in the nervous tissues of the Japanese anglerfish Lophius litulon tissues of the Japanese anglerfish Lophius litulon. *Folia Parasitol. (Praha).* **51**: 167–176.

Gachon, C.M.M., Strittmatter, M., Badis, Y., Fletcher, K.I., West, P. Van, Müller, D.G., et al. (2017) Pathogens of brown algae : culture studies of Anisolpidium ectocarpii and A . rosenvingei reveal that the Anisolpidiales are uniflagellated oomycetes. *Eur. J. Phycol.* **52**: 133–148.

Grzebyk, D., Berland, B., and Sako, Y. (1998) Phylogenetic analysis of nine species of Prorocentrum (Dinophyceae) inferred from 18S ribosomal DNA sequences, morphological comparisons, and description of Prorocentrum panamensis, sp. nov. *J. Phycol.* **34**: 1055–1068.

Hoppenrath, M., Horiguchi, T., Miyoshi, Y., Selina, M., Taylor, M.F.J.R., and Leander, B.S. (2007) Taxonomy , phylogeny , biogeography , and ecology of Sabulodinium undulatum (Dinophyceae), including an amended description of the species. *Phycol. Res.* **55**: 159–175.

Nézan, E., Tillmann, U., Bilien, G., Boulben, S., Chèze, K., Zentz, F., et al. (2012) Taxonomic revision of the dinoflagellate amphidoma caudata: Transfer to the genus azadinium (dinophyceae) and proposal of two varieties, based on morphological and molecular phylogenetic analyses. *J. Phycol.* **48**: 925–939.

Nunn, G.B., Theisen, B.F., Christensen, B., and Arctander, P. (1996) Simplicity-Correlated Size Growth of the Nuclear 28S Ribosomal RNA D3 Expansion Segment in the Crustacean Order Isopoda. *J. Mol. Evol.* **42**: 211–223.

Yamaguchi, A. and Horiguchi, T. (2005) Molecular phylogenetic study of the heterotrophic dinoflagellate genus Protoperidinium (Dinophyceae) inferred from small subunit rRNA gene sequences. *Phycol. Res.* **53**: 30–42.
