# Supplementary material for: Novel Widespread Marine Oomycetes Parasitising Diatoms, Including the Toxic Genus Pseudo-nitzschia: Genetic, Morphological, and Ecological Characterisation
Source: Front Microbiol. 2018 Dec 3;9:2918. doi: 10.3389/fmicb.2018.02918 (PMC6286980; doi:10.3389/fmicb.2018.02918)
Supplement: Supplementary file 2 [file Table_2.pdf]

Gblocks 0.91b	
Number of sequences	81
Alignment assumed to be	DNA
New number of positions	1206 (63% of the original 1896 positions)
**Parameters used**	
Minimum Number Of Sequences For A Conserved Position	41
Minimum Number Of Sequences For A Flanking Position	68
Maximum Number Of Contiguous Nonconserved Positions	8
Minimum Length Of A Block	5
Allowed Gap Positions	All
Flank positions of the 3 selected blocks	Flanks: [154 292] [308 689] [716 1398]

Table S2: Gblocks parameters used in this study
