# Supplementary material for: Novel Widespread Marine Oomycetes Parasitising Diatoms, Including the Toxic Genus Pseudo-nitzschia: Genetic, Morphological, and Ecological Characterisation
Source: Front Microbiol. 2018 Dec 3;9:2918. doi: 10.3389/fmicb.2018.02918 (PMC6286980; doi:10.3389/fmicb.2018.02918)
Supplement: Supplementary file 7 [file Image_1.pdf]

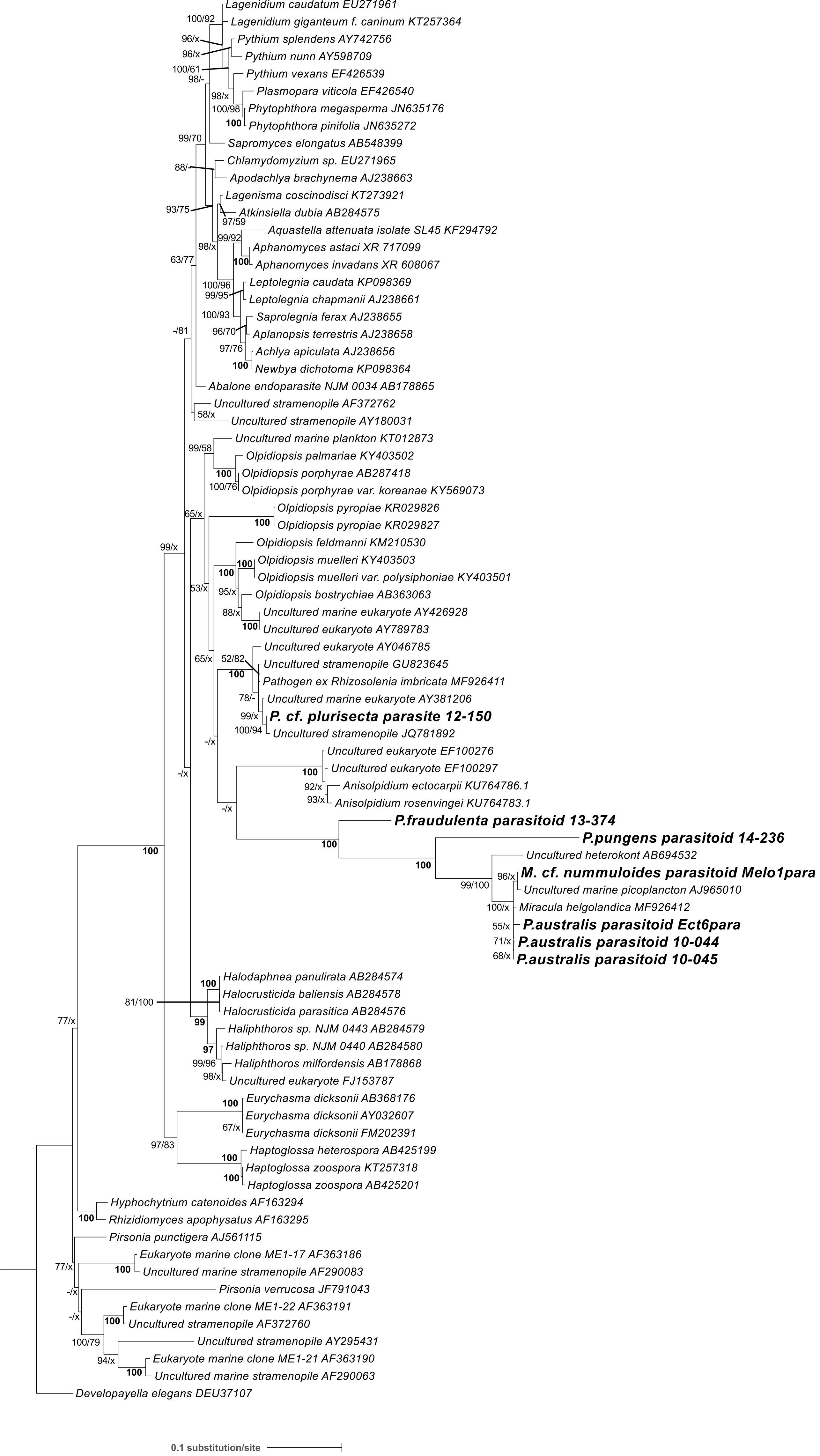

Fig S1 | Maximum Likelihood phylogenetic tree reconstruction based on partial 18S rDNA genes of the novel diatom parasitoids within the Oomycota (Stramenopila), inferred from 81 sequences on 1344 positions, before the Gblocks trimming step . New sequences are in bold and they are named according to the host species and the sample ID within the new clades OOM_1 and OOM_2 (details in Table 1). Bootstrap values (1000 replicates for ML and NJ) are shown at each node as a percentage for the 2 computational methods tested, with the following order: Maximum likelihood and Neighbour joining. X: node not supported; -: bootstrap support < 50%. Bold numbers indicate agreement on the same bootstrap value for the three methods.
