# Supplementary material for: Novel Widespread Marine Oomycetes Parasitising Diatoms, Including the Toxic Genus Pseudo-nitzschia: Genetic, Morphological, and Ecological Characterisation
Source: Front Microbiol. 2018 Dec 3;9:2918. doi: 10.3389/fmicb.2018.02918 (PMC6286980; doi:10.3389/fmicb.2018.02918)
Supplement: Supplementary file 8 [file Image_2.pdf]

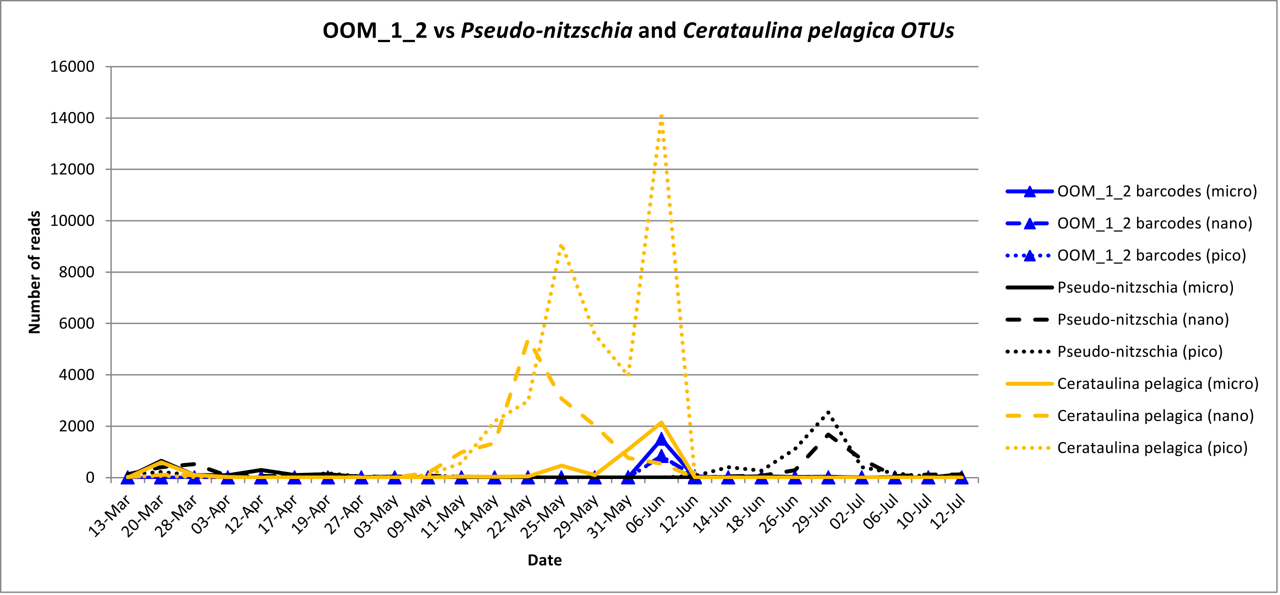

Fig S 2 | Trend of *Pseudo-nitzschia* (black) and *Cerataulina pelagica* (yellow) OTUs in relation to the barcode associated to OOM_1_2 (blue). Solid lines indicate microplankton (> 20 µm), dashed lines nano plankton (20 –3 µm) and dotted lines picoplankton (< 3 µm). Y axis indicatesthe read numbers whilst X axis indicatesdates.
