# Supplementary material for: Novel Widespread Marine Oomycetes Parasitising Diatoms, Including the Toxic Genus Pseudo-nitzschia: Genetic, Morphological, and Ecological Characterisation
Source: Front Microbiol. 2018 Dec 3;9:2918. doi: 10.3389/fmicb.2018.02918 (PMC6286980; doi:10.3389/fmicb.2018.02918)
Supplement: Supplementary file 9 [file Image_3.pdf]

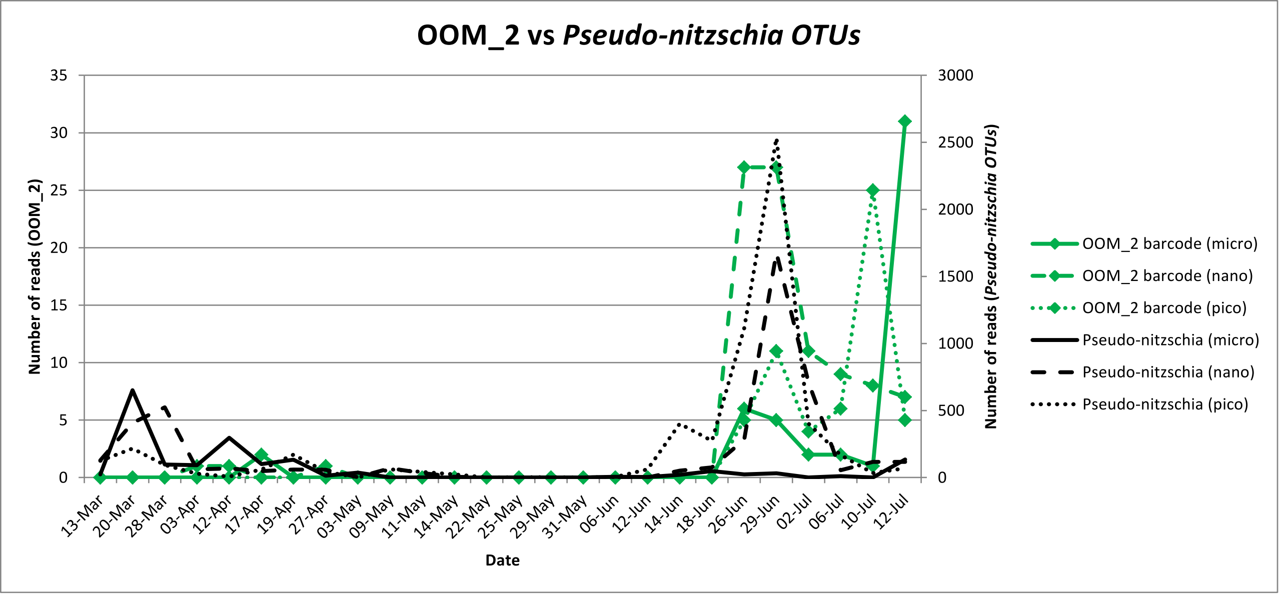

Fig. S3|Trend of *Pseudo-nitzschia*OTUs (black) in relation to the barcode associated to OOM_2 (green).Solid lines indicate microplankton (> 20 µm), dashed lines nano plankton (20 –3 µm) and dotted lines picoplankton (< 3 µm). Note that the read numbers for OOM_2 are shown on the left Y axis, whilst read numbers for *Pseudo-nitzschia*OTUs are shown on the right Y axis. X axis indicate sampling dates.
