# Supplementary material for: Novel Widespread Marine Oomycetes Parasitising Diatoms, Including the Toxic Genus Pseudo-nitzschia: Genetic, Morphological, and Ecological Characterisation
Source: Front Microbiol. 2018 Dec 3;9:2918. doi: 10.3389/fmicb.2018.02918 (PMC6286980; doi:10.3389/fmicb.2018.02918)
Supplement: Supplementary file 11 [file Image_5.pdf]

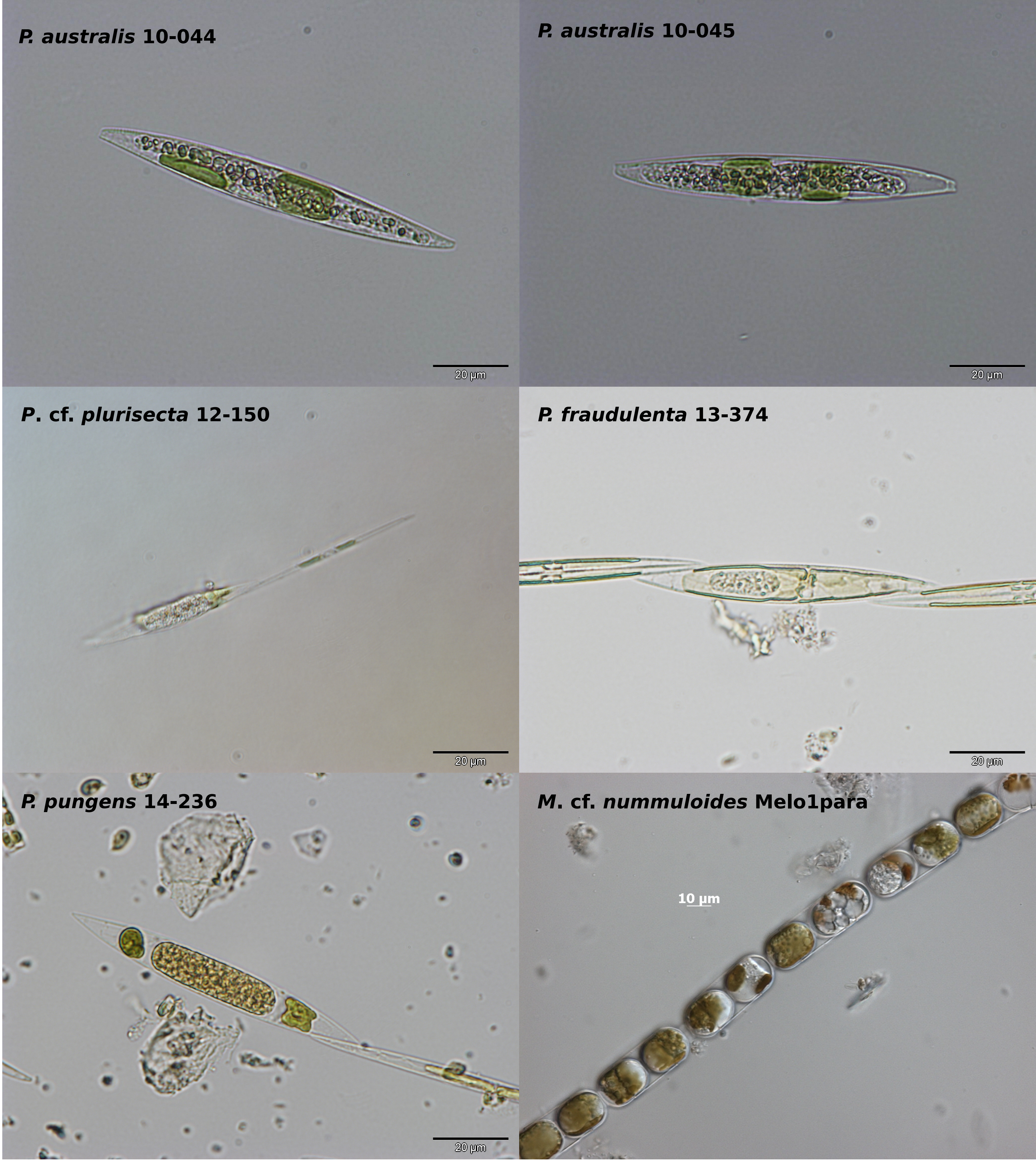

Fig.S5|Sequencedsingle cell isolates. Pictures show the single cell (or colony) sequenced to obtain the genetic marker indicated by the name of the host diatom and PCR code on the top left side. Table 1, summarizing the details for each isolate, is reported in the main text.
